# Supplementary material for: Comparative multiomics analysis of cell physiological state after culture in a basket bioreactor
Source: Sci Rep. 2022 Nov 23;12:20161. doi: 10.1038/s41598-022-24687-4 (PMC9686226; doi:10.1038/s41598-022-24687-4)
Supplement: Supplementary file 1 — Supplementary Information 1. [file 41598_2022_24687_MOESM1_ESM.zip › raw data/Transcriptomic raw data/6.Enrichment/1.GO/TE1vsTC1/DOWN/TE1vsTC1.down.GObp_DAG.pdf]

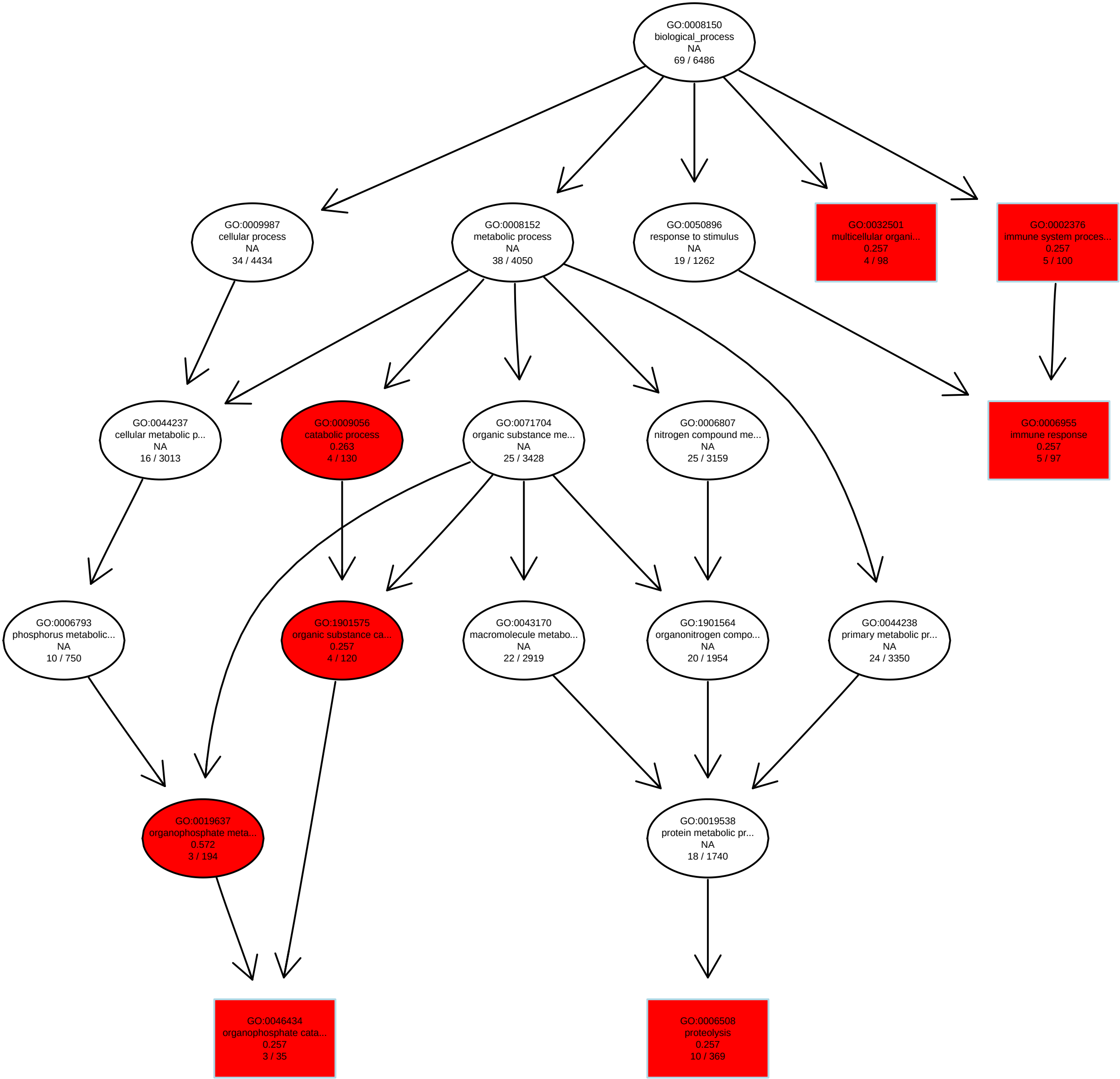

GO:0008150
biological_process
NA
69 / 6486
GO:0009987
cellular process
NA
34 / 4434
GO:0008152
metabolic process
NA
38 / 4050
GO:0050896
response to stimulus
NA
19 / 1262
GO:0032501
multicellular organi...
0.257
4 / 98
GO:0002376
immune system proces...
0.257
5 / 100
GO:0044237
cellular metabolic p...
NA
16 / 3013
GO:0009056
catabolic process
0.263
4 / 130
GO:0071704
organic substance me...
NA
25 / 3428
GO:0006807
nitrogen compound me...
NA
25 / 3159
GO:0006955
immune response
0.257
5 / 97
GO:0006793
phosphorus metabolic...
NA
10 / 750
GO:1901575
organic substance ca...
0.257
4 / 120
GO:0043170
macromolecule metabo...
NA
22 / 2919
GO:1901564
organonitrogen compo...
NA
20 / 1954
GO:0044238
primary metabolic pr...
NA
24 / 3350
GO:0019637
organophosphate meta...
0.572
3 / 194
GO:0019538
protein metabolic pr...
NA
18 / 1740
GO:0046434
organophosphate cata...
0.257
3 / 35
GO:0006508
proteolysis
0.257
10 / 369
